# Intact Parathyroid Hormone (iPTH) Assay: An Early Approach for Bone Health Assessment in Chronic Renal Failure

**DOI:** 10.7759/cureus.72510

**Published:** 2024-10-27

**Authors:** Sweta Kumari, Prit P Singh, Dilip Kumar, Naresh Kumar, Santosh Kumar, Ravi Shekhar

**Affiliations:** 1 Biochemistry, Indira Gandhi Institute of Medical Sciences, Patna, IND; 2 Nephrology, Indira Gandhi Institute of Medical Sciences, Patna, IND; 3 Medicine, Indira Gandhi Institute of Medical Sciences, Patna, IND

**Keywords:** chronic kidney disease, diabetes, egfr, hyperparathyroidism, hypertension, intact parathyroid hormone, kidgo classification 2024

## Abstract

Background: Chronic kidney disease (CKD) is a global public health burden with significantly increasing mortality. Chronic kidney disease leads to various complications, including anemia, cardiovascular complications, salt and water retention, metabolic acidosis, electrolyte disorders, and chronic kidney disease-mineral and bone disorder (CKD-MBD), amongst which CKD-MBD is a particularly important complication that develops early.

Materials and methods: In the present study, various biochemical analytes were assessed for their significance in monitoring the CKD-MBD in different stages of CKD. Only biochemistry markers were assessed in the present study, as X-rays and other radiological markers give positive results in the very late stages of CKD.

Results: The level of intact parathyroid hormone (iPTH) was raised or highly normal from the very early stages of chronic renal failure (CRF), and the differences in progressive stages were highly significant p<0.05. The correlation of iPTH with different biochemistry test parameters of CRF, such as creatinine, estimated glomerular filtration rate (eGFR), vitamin D, calcium, and inorganic phosphate, was also checked to determine its efficacy in the detection of CKD-MBD and, consequently, in preventing the complication of CKD. Parathyroid hormone (PTH) showed a significant positive correlation with creatinine in Stages 4 and 5 (p<0.05 in both stages). It was also observed that with the increase in the PTH level, there was a decrease in the estimated glomerular filtration rate in both Stage 4 and 5 patients.

Conclusion: The level of iPTH was significantly increased from Stage 2 onwards; hence, it can be used as a diagnostic marker of CKD-MBD from the initial stages. Subsequent to diagnosis, the progression of CKD-MBD can be prevented by choosing the appropriate intervention from the wide array of treatment options available.

## Introduction

Chronic kidney disease (CKD) is emerging as a global public health burden. It is associated with significant morbidity and has continued to rise in rank among the leading causes of death [1]. Approximately 850 million people worldwide were estimated to have kidney disease in the year 2019 [2]. In India, the prevalence of CKD is 800 per million of the population, and the incidence of end-stage renal failure is estimated to be 200 per million of the population [3]. There are various contributory conditions, among which diabetes and hypertension (HTN) account for two-thirds of cases of CKD [4]. Progressive CKD leads to several complications, including anaemia, cardiovascular complications, chronic kidney disease-mineral and bone disorder (CKD-MBD), salt and water retention, metabolic acidosis, electrolyte disorders, and uraemic symptoms [5]. Chronic kidney disease-mineral and bone disorder, previously known as renal osteodystrophy, is a well-known complication of CKD and is characterized by altered metabolism of calcium, phosphate, parathyroid hormone (PTH), and vitamin D.

The diagnosis of CKD-MBD relies on these biochemistry markers, as X-rays and other bone imaging methods detect skeletal changes in the late stages. The intact parathyroid hormone (iPTH) assay is the standard method for measuring the level of PTH in CKD patients and continues to be the most frequently used marker for correlating the clinical diagnosis of renal osteodystrophy or bone disease in CKD cases [6]. The primary translational product of the PTH is 115-amino acid long pre-pro-PTH. Both the pre- and pro-(6 amino acid long) sequences are enzymatically cleaved to produce 84 amino acid long iPTH, which is secreted into the bloodstream and undergoes extensive proteolytic modifications. In contrast with its degradation products, the concentration of iPTH is less affected by the glomerular filtration rate (GFR) and reflects the biologically active portion of the hormone [7,8].

Hyperparathyroidism secondary to CKD is an overproduction of PTH caused by changes that occur in bone and mineral metabolism owing to decreased kidney function. The initial changes that usually occur with declining kidney function involve the deficiency of active vitamin D and retention of phosphorus by the remaining functional nephrons. Both changes stimulate PTH synthesis and secretion, which promotes phosphorus excretion from the kidneys and the reabsorption of calcium from the renal tubules [9]. Hyperparathyroidism is characterized by atypical bone histology, bone pain, and fractures among patients with either primary or secondary hyperparathyroidism (SHPT) [10]. Secondary hyperparathyroidism is also associated with cardiovascular disease and is one of the most common causes of death among CKD patients. Many recent observational studies have reported increased cardiovascular risk in CKD patients associated with even a slight increase in the PTH level. The most frequently observed cardiac complication in CKD is left ventricular hypertrophy, which is often associated with poor perfusion, myocardial fibrosis, and cell death [11]. In SHPT, hyperphosphatemia occurs, leading to vascular and soft tissue calcifications, which are strong predictors of cardiovascular mortality in CKD patients [12].

If early and appropriate intervention could be taken in the early stages of CKD, skeletal and cardiac complications due to mineral disturbances could be prevented or delayed. Hence, biochemical markers, such as the PTH level, should be monitored for the detection of CKD-MBD. With this in mind, this study aims to estimate the serum iPTH in different stages of CKD to assess its efficacy as an early marker of CKD-MBD. Additionally, the correlation of iPTH with different test parameters of chronic renal failure (CRF), such as creatinine, estimated glomerular filtration rate (eGFR), vitamin D, calcium, and inorganic phosphate, is also assessed to confirm the utility of this biomarker in the detection of CKD-MBD and, consequently, in preventing the complication of altered bone mineral metabolism in CKD.

## Materials and methods

This cross-sectional study was conducted from November 2022 to May 2024 in the Department of Biochemistry with the Association of Department of Nephrology, Indira Gandhi Institute of Medical Sciences (IGIMS), Patna, India. Ethical consent was obtained from the Institutional Ethical Committee before starting this study (732/IEC/IGIMS/2022). The sample size was calculated for the correlation bivariate normal model using G*Power software (Version 3.1.9.7, Heinrich-Heine-Universität, Düsseldorf, Germany) with α = 0.05, β = 0.8, effect size = 0.1, and two-tailed. The sample size was estimated to be 782; assuming a 10% drop rate, we considered 860 subjects for the study, out of which 855 subjects were enrolled. All male and female patients diagnosed with CKD who were over 18 years of age and attending the Department of Nephrology and General Medicine (both outpatient department and inpatient department) with HTN, diabetes mellitus, or both were included in the study. Clinical data were documented, including a detailed history of the patients, after obtaining informed written consent. The exclusion criteria were patients with a known history of any phosphate wasting disorder (e.g., X-linked hypophosphataemic rickets), pregnancy, inability to consent, and enrolment in other studies. Venous blood samples were collected at the sample collection area in a vacutainer with prescribed antiseptic precautions, and the samples were then transported by a pneumatic transport system to the laboratory, where they were centrifuged at 3,000 rpm for 10 minutes. The serum was then separated and analyzed for different parameters. Serum creatinine, inorganic phosphorus, and calcium were measured using a chemistry analyzer, Beckman Coulter AU 5800 (Beckman Coulter, Inc., Brea, CA), and serum vitamin D and serum iPTH were measured using a chemiluminescence micro-particle immunoassays instrument, Architect i-2000SR (Abbott, Chicago, IL). The reagents were procured from the same manufacturer. The samples were analyzed after a routine multilevel quality check for all parameters.

Kidney function is primarily assessed by the GFR. A normal GFR is 120 ml/min/1.73 m^2^, though this varies with age, sex, race, and other factors. In the Kidney Disease Improving Global Outcome (KDIGO) 2024 guidelines, CKD is described as the presence of kidney damage or an eGFR of less than 60 ml/min/1.73 m^2^ continuing for three months or more, irrespective of the cause [4, 13]. In addition, CKD is defined as abnormalities in kidney structure or function that present for a minimum of three months and have health implications [13].

The criteria for evaluating CKD (any of the following present for a minimum of three months) are as follows: A) markers of kidney damage (one or more), which include 1) albuminuria (albumin-creatinine ratio (ACR) ≥30 mg/g (≥3 mg/mmol)), 2) urine sediment abnormalities, 3) persistent hematuria, 4) electrolyte and other abnormalities due to tubular disorders, 5) abnormalities detected by histology, 6) structural abnormalities detected by imaging, and 7) history of kidney transplantation; B) decreased GFR-GFR≤60mL/min/1.73m^2^(GFR categories G3a-G5).

The eGFR was conducted using the CKD-EPI equation 2021. The GFR categories were assigned as follows: Grade 1: 90 ml/min/1.73 m^2^ or above; Grade 2: between 60 and 89; Grade 3a: between 45 and 59; Grade 3b: between 30 and 44; Grade 4: between 15 and 29; Grade 5: less than 15 ml/min/1.73 m^2^.

In this study, the normality of the data was checked using the Cramér-von Mises test. Parametric data are expressed in terms of mean± SD, and non-parametric data are expressed in terms of median (interquartile range (IQR)). The results of the continuous variable are expressed as mean ±SD (min-max), and the results of the categorical variable are expressed as a number (%). The Spearman’s rank correlation coefficient was determined to identify any associations between the two groups. The significance of the study was assessed by a p-value ≤0.05. SPSS 16.0 software (SPSS Inc., Chicago, IL) and Microsoft Excel (Microsoft Corp., Redmond, WA) were used for the statistical analysis of the data.

## Results

The study group was composed of 855 cases, divided into six groups according to KDIGO classification 2024. Table 1 shows the maximum number of cases in Grade 5 and the minimum number of cases in Grade 2 (331 and 75 cases, respectively). The observed distribution was composed of 69.7% male subjects and 30.3% female subjects.

**Table 1:** Distribution of the subjects according to their study history CKD: chronic kidney disease; DM: diabetes mellitus; HTN: hypertension

	CKD Grade 1	CKD Grade 2	CKD Grade 3A	CKD Grade 3B	CKD Grade 4	CKD Grade 5
Total no. of cases	102	75	49	99	199	331
Number of diabetic cases (DM%)	30 (29.41)	23 (30.66)	19 (38.77)	40 (40.40)	78 (39.19)	117 (35.34)
Number of hypertensive cases (HTN%)	36 (35.29)	28 (37.34)	23 (46.93)	38 (38.38)	85 (42.79)	156 (47.12)
Number of both DM and HTN cases (both%)	36 (35.30)	24 (32)	7 (14.28)	21 (21.21)	36 (18.09)	58 (29.14)

As shown in Table 2, there was a significant difference (p-value<0.05) in the level of all biochemical test parameters (PTH, creatinine, calcium, inorganic phosphate, and eGFR) among the different groups, except for vitamin D, which didn’t differ significantly among the different grades. Grade 1 had the lowest age with a mean of 31.4 ± 11.6 years. The highest age group was Grade 3A, with a mean of 56.1 ±14.0 years.

**Table 2:** Median level of biochemical test parameters in different grades of CRF IQR: interquartile range; PTH: parathyroid hormone; eGFR: estimated glomerular filtration rate; CRF: chronic renal failure Normal values and units: intact parathyroid hormone (iPTH): 15-68pg/mL; creatinine: 0.5-1.12mg/dL; eGFR: >60 ml/min/1.73 m^2^; vitamin D: >30ng/mL; inorganic phosphate: 2.5-4.5mg/dL

Parameters	Grade	N	Median (IQR)	χ2	df	P-value*
PTH	1	102	65.80 (47.3)	503.93	5	<0.05
	2	75	78.30 (55.1)			
	3A	49	87.307 (5.25)			
	3B	99	120.20 (98.7)			
	4	199	247.45 (245.2)			
	5	331	470.30 (396.7)			
Creatinine	1	102	0.82 (0.27)	772.04	5	<0.05
	2	75	1.1 (0.33)			
	3A	49	1.5 (0.33)			
	3B	99	2.11 (0.35)			
	4	199	3.18 (0.99)			
	5	331	7.25 (4.62)			
eGFR	1	102	112 (28.25)	790.65	5	<0.05
	2	75	74 (19)			
	3A	49	51 (6)			
	3B	99	35 (8)			
	4	199	21 (7)			
	5	331	8 (5)			
Vitamin D	1	102	17.5 (8.72)	13.48	5	0.019
	2	75	20 (12)			
	3A	49	19.3 (13.55)			
	3B	99	21.1 (13.9)			
	4	199	18.65 (15.5)			
	5	331	17.1 (14)			
Calcium	1	102	9.8 (0.53)	220.04	5	<0.05
	2	75	9.6 (0.8)			
	3A	49	9.3 (.0.75)			
	4B	99	9.4 (0.8)			
	4	199	9.2 (0.9)			
	5	331	8.6 (1.2)			
Inorganic phosphate	1	102	3.85 (1.1)	369.10	5	<0.05
	2	75	3.6 (0.9)			
	3A	49	3.8 (1.2)			
	3B	99	3.8 (0.9)			
	4	199	4.3 (1.1)			
	5	331	6 (2.5)			

**Figure 1:**
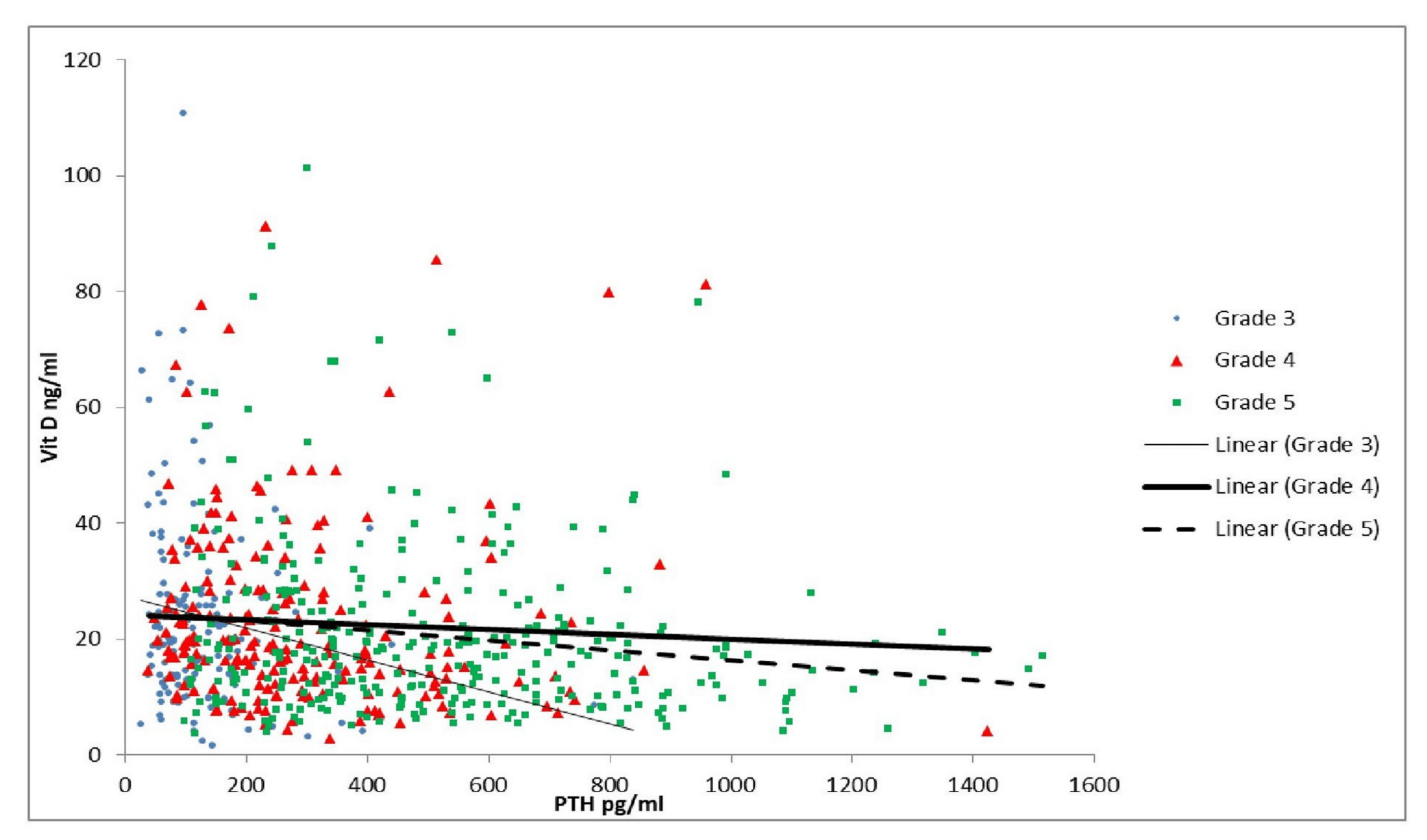
Correlation of parathyroid hormone (PTH) with vitamin D (Vit D) in chronic kidney disease (CKD) Grades 3, 4, and 5

**Figure 2:**
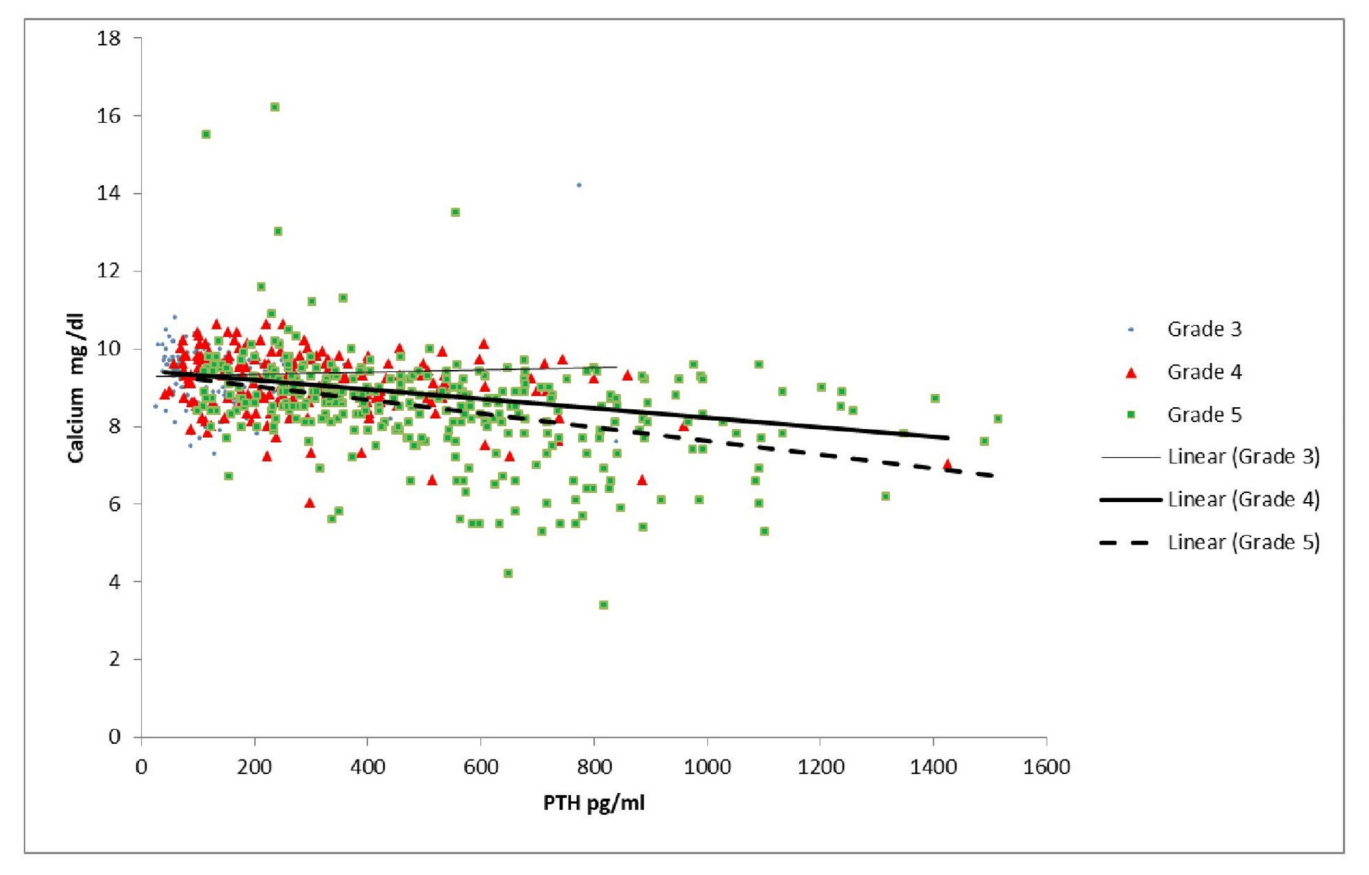
Correlation of parathyroid hormone (PTH) with calcium in chronic kidney disease (CKD) Grades 3, 4, and 5

**Figure 3:**
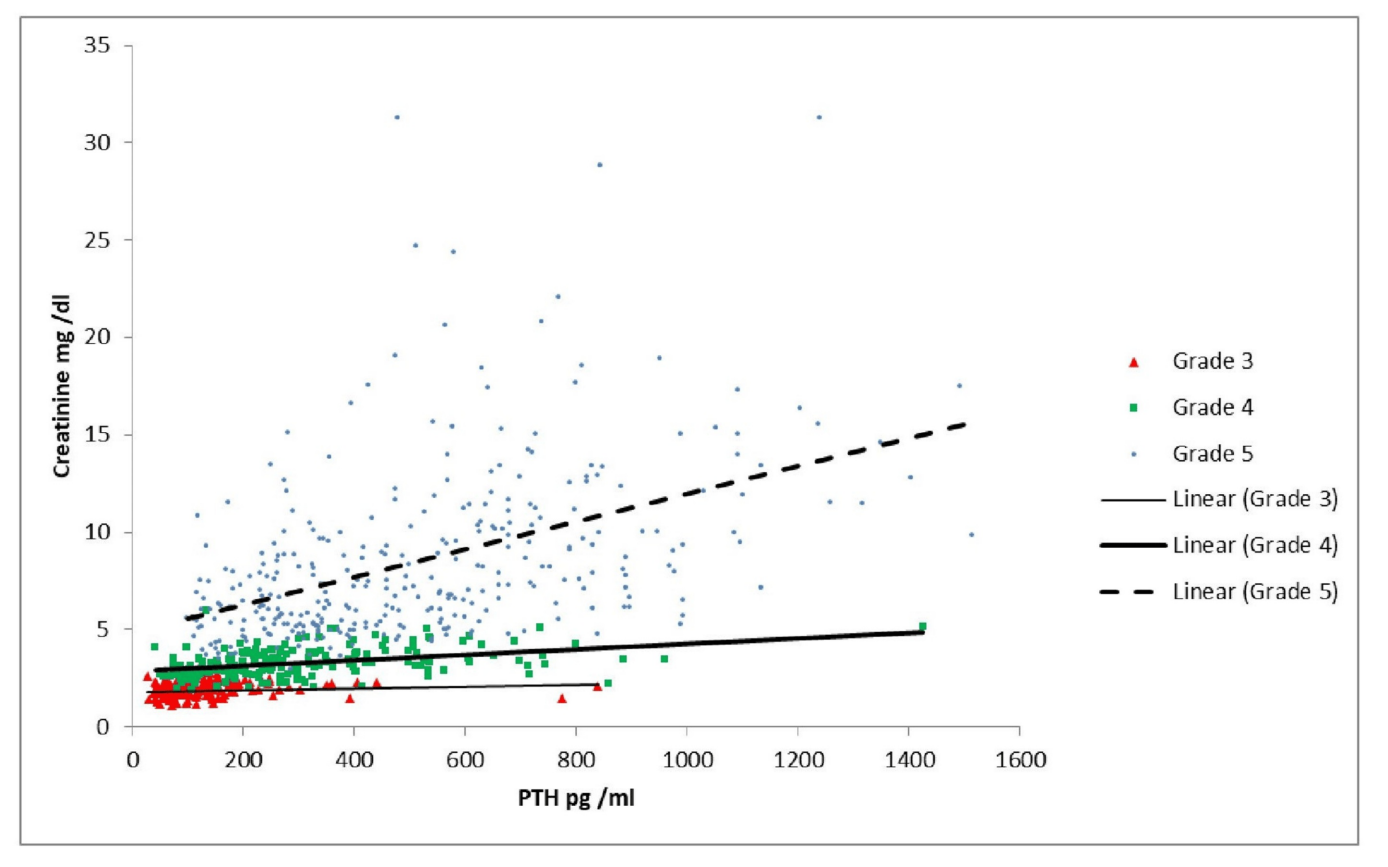
Correlation of parathyroid hormone (PTH) with creatinine in chronic kidney disease (CKD) Grades 3, 4, and 5

**Figure 4:**
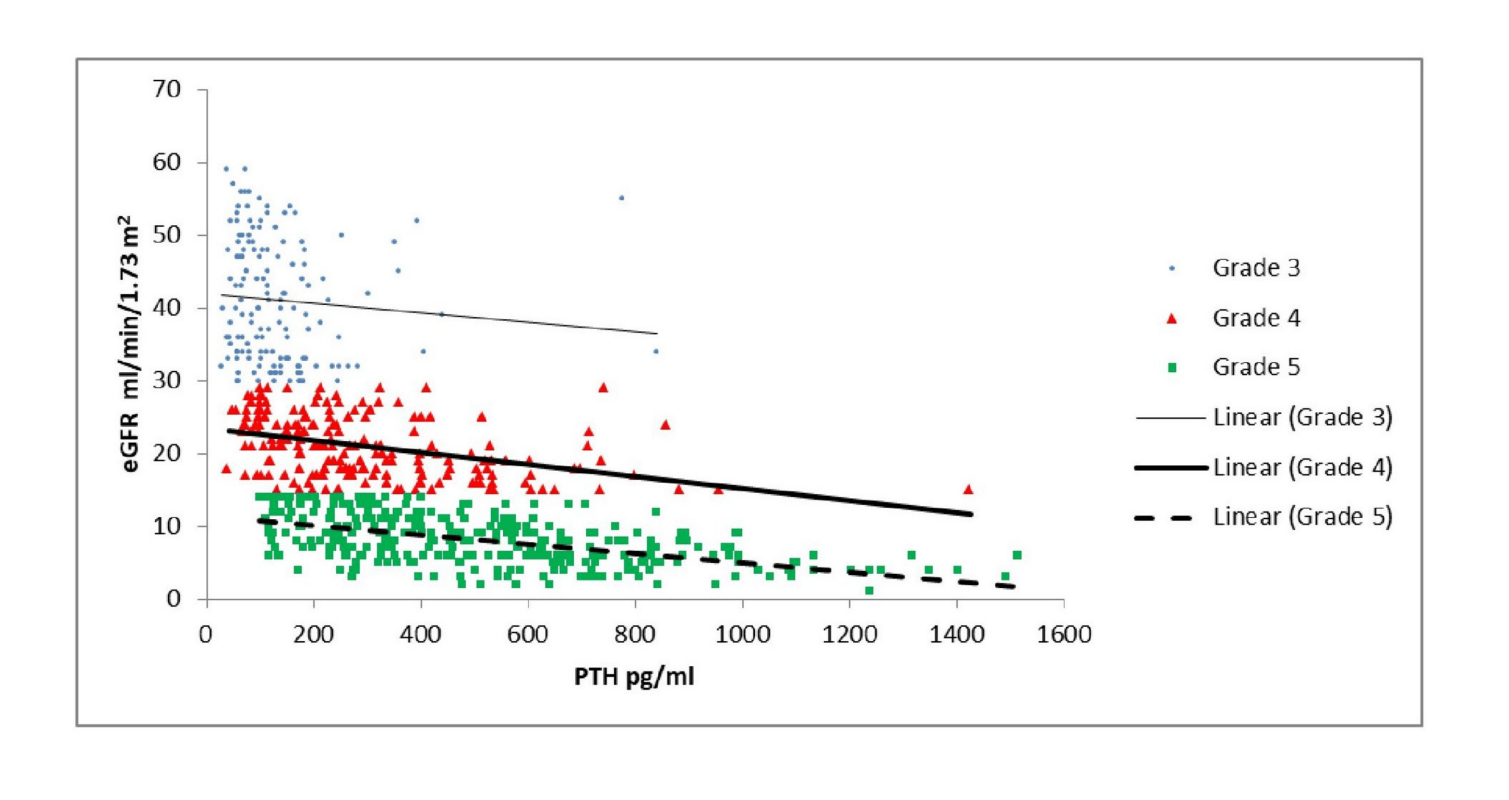
Correlation of parathyroid hormone (PTH) with estimated glomerular filtration rate (eGFR) in chronic kidney disease (CKD) Grades 3, 4, and 5

**Figure 5:**
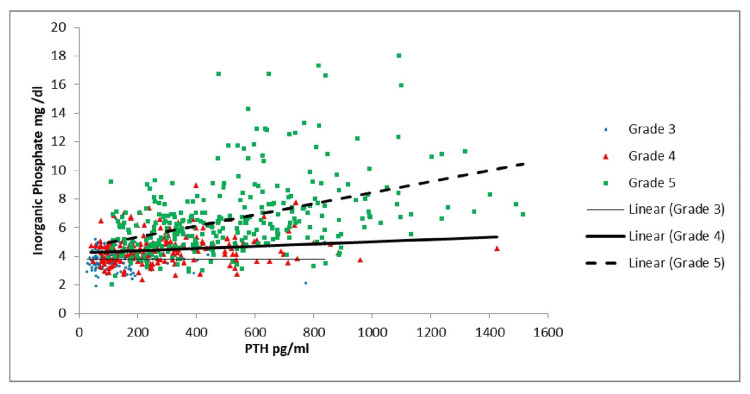
Correlation of parathyroid hormone (PTH) with inorganic phosphate in chronic kidney disease (CKD) Grades 3, 4, and 5

As shown in Table 3 and Figure 1, the correlation of vitamin D with PTH was significant in Grades 2, 3B, and 5. As Table 3 and Figure 2 indicate, there was a similar correlation between calcium and PTH, which was significant in Grades 3B and 5. Per Table 3 and Figure 3, there was a significant positive correlation between PTH and creatinine in Grades 4 and 5 patients. As shown in Table 3 and Figure 4, it was also observed that with the increase in PTH level, there was a decrease in eGFR in both Grades 4 and 5 patients (ρ = -0.437, p <0.05; ρ = -0.556, p <0.05). However, these associations were weak in nature. The level of inorganic phosphate was positively correlated to PTH only in Grade 5 (Table 3 and Figure 5).

**Table 3:** Correlation of PTH with creatinine, eGFR, vitamin D, calcium, and inorganic phosphate in the different stages of CRF PTH: parathyroid hormone; eGFR: estimated glomerular filtration rate; CRF: chronic renal failure

Grade		Spearman’s rank correlation coefficient(ρ)	p-value
1	PTH vs creatinine	–0.021	0.832
	PTH vs eGFR	–0.088	0.380
	PTH vs vitamin D	–0.131	0.189
	PTH vs calcium	–0.094	0.348
	PTH vs inorganic phosphate	–0.120	0.229
2	PTH vs creatinine	0.129	0.268
	PTH vs eGFR	–0.114	0.330
	PTH vs vitamin D	–0.331	0.004
	PTH vs calcium	–0.067	0.569
	PTH vs inorganic phosphate	–0.106	0.365
3A	PTH vs creatinine	0.231	0.111
	PTH vs eGFR	–0.181	0.214
	PTH vs vitamin D	–0.151	0.300
	PTH vs calcium	–0.201	0.166
	PTH vs inorganic phosphate	–0.153	0.295
3B	PTH vs creatinine	0.171	0.091
	PTH vs eGFR	–0.131	0.196
	PTH vs vitamin D	–0.206	0.040
	PTH vs calcium	–0.273	0.006
	PTH vs inorganic phosphate	0.086	0.397
4	PTH vs creatinine	0.425	<0.05
	PTH vs eGFR	–0.437	<0.05
	PTH vs vitamin D	–0.151	0.300
	PTH vs calcium	–0.201	0.166
	PTH vs inorganic phosphate	–0.153	0.295
5	PTH vs creatinine	0.514	<0.05
	PTH vs eGFR	–0.556	<0.05
	PTH vs vitamin D	–0.143	0.009
	PTH vs calcium	–0.418	<0.05

## Discussion

Patients with CKD are asymptomatic, particularly in early grades; therefore, screening may be important in the early detection of the disease. Appropriate screening, early diagnosis, and management are necessary for preventing CKD-associated adverse outcomes, including cardiovascular disease, end-stage renal disease, and death [14-17]. The National Kidney Foundation has developed a kidney profile test that measures both serum creatinine for estimating eGFR and spot urinary ACR. A risk-based approach to screening is recommended in high-risk groups, individuals older than 60 years, and individuals with a history of diabetes and HTN [18]. Assessing renal function in individuals with type 2 diabetes of more than five years duration is essential, as diabetic nephropathy constitutes the main cause of CKD, ultimately resulting in end-stage renal disease [19]. In patients presenting with advanced grades of renal impairment, there is a need to strengthen strategies for CKD identification and prevention.

Patients with CKD have progressively declining GFR and develop mineral metabolic disturbances, along with other complications. Decreased renal function hinders the kidneys’ ability to maintain fluid and electrolyte homeostasis. The decline in renal function results in hypocalcemia, hyperphosphatemia, and reduced calcitriol levels, stimulating PTH synthesis and secretion while promoting parathyroid gland hyperplasia, leading to secondary hyperparathyroidism [7]. The damaged kidney is unable to excrete its phosphorus load properly; in addition, conversion to active metabolite calcitriol is hindered, which leads to compensatory SHPT. The mineral and endocrine functions that are disturbed in CKD are vital in the regulation of bone remodelling. As a result, bone abnormalities, such as altered remodelling and loss of bone volume, are common in patients with CKD Grades 3 to 5.

In the present study, the PTH level progressively increased with the advancing stages of CKD, similar to that observed by Natikar et al. in 2020. The authors compared the level of PTH in patients of various stages of CKD. As the disease progressed, there was a progressive increase in the PTH level [9]. Derangements in mineral metabolism are also associated with cardiovascular disease and various other causes of mortality. In patients on dialysis, cardiovascular mortality rates are 10 to 500 times higher than the general population [7]. Patients with CKD Grades 3 to 5 typically have a substantially higher risk of CVD than patients with Grades 1 to 2 CKD [20]. Cardiac valve calcification can lead to cardiac conduction dysfunction, myocardial ischemia or infarction, valve insufficiency, congestive cardiac failure, and other complications, increasing the risk of cardiovascular death. Hyperphosphatemia is an important cause of increased vascular calcification in patients with CKD, which also leads to an increase in mortality. The exact mechanism of calcification caused by CKD, however, has yet to be identified. Small studies have shown that renal function loss is faster and the incidence of valve calcification is higher in patients with CKD Grade 5 hyperphosphatemia without dialysis, and the serum phosphate level can be used as an independent predictor of the total calcification score [21].

In the present study, serum PTH had a linear negative correlation with serum calcium in Grade 5 (p<0.05,ρ= -0.418) but a linear positive correlation with serum inorganic phosphate (p<0.0.001,ρ= 0.471) in CKD Grade 5. A similar result was observed by Arora et al. in 2018, who showed that the serum PTH had a linear negative correlation with serum calcium (p<0.01,ρ=-0.421) but a linear positive correlation with serum inorganic phosphate (p<0.0.05,ρ=0.378) in CKD Grade 5 [22]. In the authors’ study, the serum PTH had a linear negative correlation with serum calcium in Grade 3b (p = 0.006, ρ = -0.273) of CKD. A similar result was noted by Carneiro Dias et al. in 2020, who found that serum PTH had a linear negative correlation with serum calcium in Grade 3 (ρ = -0.23) [23]. In addition, in this study, PTH had a linear negative correlation with serum 25-hydroxy vitamin D in all stages, and this was significant in stages 3B, 4, and 5 of CKD. Interstitial fibrosis and tubular atrophy also progress with declining renal function; this leads to the onset and further progression of mineral bone disorder. Mineral and bone disorder progresses with declining renal function; thus, a high PTH level correlates with declining renal function or an advancing stage of CKD. The same is rectified in our study, which is why there is a strong correlation. A comparable result was observed by Restrepo Valencia et al. in 2016, who found that PTH had a linear negative correlation with serum 25-hydroxy vitamin D (p =.000 ρ = -0.193) in CKD Grades 2 to 5 [24].

Patients with CKD Grades 4 and 5 are at an 8.6 times higher risk of having increased serum iPTH levels, and the prevalence of SHPT is amplified as the stage of CKD increases. As shown in Table 3, in the present study, iPTH was strongly correlated with creatinine in Grade 4 (p = <0.05, ρ = 0.425) and Grade 5 (p = <0.05, ρ = 0.514). The iPTH was also strongly negatively correlated with eGFR in Grade 4 (p = <0.05, ρ = 0.-437) and Grade 5 (p = <0.05, ρ = -0.556). In addition, as shown in Table 1, the level of iPTH progressively increased in the different stages of CRF. Hence, serum iPTH is a reliable marker for assessing SHPT in patients with CKD. Secondary hyperparathyroidism is accompanied by mortality and morbidity in CKD. It can occur in earlier stages of CKD, and through simple routine tests of serum iPTH, can be diagnosed earlier, allowing proper treatment to be initiated. Delayed diagnosis leads to resistant SHPT, which persists even after renal transplantation and accounts for cardiovascular mortality and morbidity [25].

A convenient and cost-effective method for the detection of SHPT in CRF is needed, as there are various preventive and therapeutic approaches that could be exploited to halt the development of CKD-MBD. These include both medicinal and surgical approaches to decrease the level of PTH. The medicinal approach includes vitamin D and active vitamin D, calcium mimetics, phosphate binder, and local injection of alcohol or active vitamin D derivatives. Surgical treatments include sub-total parathyroidectomy or total parathyroidectomy with immediate auto-transplantation [26].

The limitation of the study was the estimation of 25-hydroxy vitamin D rather than 1,25-dihydroxy vitamin D. This study is hospital-based and single-centric, but the implication can be improved by performing a multi-centric study. The newer markers (fibroblast growth factor 23 levels and a-Klotho) should be evaluated in the different stages of CKD to monitor prognosis and the outcome of the disease [26].

## Conclusions

Chronic kidney disease is associated with many complications, including mineral and bone disease, which begins early. The disease could be diagnosed by the biochemical marker PTH because all the radiological investigations, including X-ray and other bone imaging techniques, provide a positive result in the later stages of CRF. Parathyroid hormone is the ideal marker for monitoring SHPT in CRF, as it begins increasing in the initial stages. Once detected, the progression of CKD-MBD can be halted by choosing the appropriate intervention from the wide array of treatment options available.
